# BACE1 Inhibitors for Alzheimer’s Disease: The Past, Present and Any Future?

**DOI:** 10.3390/molecules27248823

**Published:** 2022-12-12

**Authors:** Firas H. Bazzari, Amjad H. Bazzari

**Affiliations:** 1 Faculty of Pharmacy, Jerash University, Al-Urdon St., Jerash 26150, Jordan; 2 Department of Basic Scientific Sciences, Faculty of Arts & Sciences, Applied Science Private University, Amman 11931, Jordan

**Keywords:** Alzheimer’s disease, BACE1, amyloid cascade hypothesis, small molecules, clinical trials, AD pathophysiology, risk factors

## Abstract

Alzheimer’s disease (AD) is the most prevalent neurodegenerative disorder and the most common cause of dementia in the elderly. The complexity of AD has hindered the development of either a cure or a disease-modifying therapy to halt the disease progression. Numerous hypotheses were presented in order to explain the mechanisms underlying the pathogenesis of AD. Introduced in 1992, the “Amyloid Cascade Hypothesis” had a huge impact on the field and inspired the rise of various drug candidates, especially amyloid-beta (Aβ)-directed drugs; including beta-site amyloid precursor protein cleaving enzyme 1 (BACE1) inhibitors. Adopted by a number of pharmaceutical companies, the development of BACE1 inhibitors has gained momentum in the past decade with promising results from experimental and early clinical-phase studies. Nevertheless, nearly all BACE1 inhibitors failed in later phases of clinical trials, due to safety and/or efficacy issues, and others were discontinued early in favor of second-generation small-molecule candidates. This paper aims to provide a comprehensive review of all BACE1 inhibitors to ever reach clinical trials, and we discuss the challenges and different perspectives on whether BACE1 inhibitors are to be reconsidered or revitalized in the future.

## 1. Introduction

Alzheimer’s disease (AD) is a debilitating neurodegenerative disorder and considered the major cause of dementia in the elderly [1]. According to the 2022 world Alzheimer’s report, there are currently over 55 million individuals diagnosed with AD worldwide with an estimate that the number may exceed 130 million by the year 2050 [2]. The report has also highlighted the importance of early detection and diagnosis of AD, with estimates that as much as 75% of individuals with dementia are not diagnosed globally [2].

For well over a century since the first diagnosed case with AD [3], substantial efforts for understanding the disease pathophysiology, contributing risk factors, progression and treatment have been undertaken with a boost in scientific research aided by the introduction of biochemical analysis in the 70s and 80s of the past century [4]. Early discoveries have successfully introduced the cholinergic hypothesis for AD, where reduced cholinergic activity in the brain was suggested to be the major cause of AD, and formed the scientific base for the currently approved drugs for AD [5]. However, and despite all efforts over the past decades, AD still lacks either a cure or a disease modifying therapy to slow down the disease progression, and the currently available pharmacological options are limited to acetylcholinesterase (AchE) inhibitors and the N-methyl-D-aspartate (NMDA) receptor blocker memantine that may only provide modest symptomatic management in AD patients with no capability to alter the disease progression [6]. Furthermore, the wide array of risk factors found to be associated with AD adds on to the complexity of the disease, ranging from constitutional risk factors (e.g., cardiovascular diseases and genetic predisposition) to newly identified ones (e.g., vitamin D deficiency and thyroid imbalance), necessitating further investigations to elucidate the mechanisms linking them to the pathogenesis of AD [7,8,9].

Later into the process, the focus has shifted towards examining the histopathological hallmarks of the disease, namely the extracellular amyloid-beta (Aβ) plaques and intracellular neurofibrillary tangles [10]. These investigations have begun a new era in the discovery and development of newer medications to treat or, at least, halt the disease progression, which followed the steps of the “Amyloid Cascade Hypothesis” published back in 1992 [11]. The hypothesis had a huge impact on AD research and numerous agents were developed and advanced into human clinical trials, targeting the molecular basis of the hypothesis. Among these are inhibitor of the beta-site amyloid precursor protein cleaving enzyme 1 (BACE1), also referred to as beta-secretase 1, which is considered a vital contributor in the formation of Aβ plaques [12].

This paper provides an overview of the molecular basis of the “Amyloid Cascade Hypothesis” with a focus on the central role of Aβ, BACE1 and associated signaling pathways, and a comprehensive examination of BACE1 inhibitors by tracking their timeline from early rise in experimental studies to later fall in clinical trials. Lastly, we discuss future perspectives regarding the fate of the hypothesis and BACE1 inhibitors.

## 2. The “Amyloid Cascade Hypothesis” and Beyond

**Figure 1:**
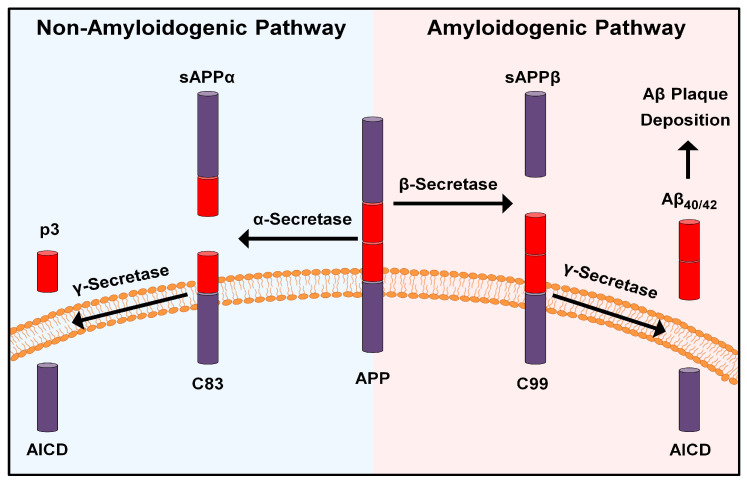
Illustration of the non- and amyloidogenic pathways.

The original hypothesis provided some insights into the mechanistic role of BACE1 in the pathogenesis of AD [11]. The amyloid precursor protein (APP) is an integral transmembrane protein suggested to mediate a number of biological functions, such as the development of the nervous system, formation of neuromuscular junctions, synaptic plasticity, axonal growth and others [13]. Under physiological conditions, APP undergoes proteolytic cleavage initially via the alpha (α)-secretase enzyme, that cuts APP within the Aβ domain, followed by gamma (γ)-secretase, eventually resulting in the release of soluble APP-α molecules, which are found to play some beneficial effects, including coordination of cellular responses and other proliferative effects [14]. This cleavage sequence has been classically referred to as the “Non-Amyloidogenic Pathway” (Figure 1).

On the other hand, the sequential activity of BACE1 and γ-secretase on APP results in the formation of Aβ isoforms; namely the insoluble Aβ_42_. This isoform is found in higher concentrations in AD patients and is more prone to aggregation and deposition in the brain resulting in the development of Aβ plaques (i.e., a major hallmark of AD) [15]. Accordingly, this proteolytic sequence has been referred to as the “Amyloidogenic Pathway” and has led to the suggestion that abnormal BACE1 activity is responsible for the pathogenesis of AD and highlighted the potential of BACE1 as a promising target for drug development to counteract AD progression [16].

Indeed, abnormal BACE1 activity is a key factor in AD pathogenesis, and this can be clearly observed in patients with trisomy 21 (i.e., Down Syndrome), where a marked increase in BACE1 is found to be the key contributor in the development of early-onset familial AD in this group [17]; thus, further supporting the potential of BACE1 as a target for AD. Nevertheless, familial AD represents only a minority of all AD cases (< 5%) [18], while the vast majority (i.e., sporadic AD) still lack a clear picture regarding the exact molecular pathways underlying the disease. This is in addition to a long list of non-genetic risk factors associated with AD, thus adding further to its complexity. However, subsequent research has provided more insights into the molecular mechanisms meditating AD pathogenesis, highlighting the cross-talk between the formation of AD hallmarks and multiple other pathways; including but not limited to, neuro-inflammation [19], insulin signaling [20] oxidative stress [21] autophagy [22] tauopathies [23] and disrupted neuromodulatory control [24] among other pathways found to influence Aβ production.

**Figure 2:**
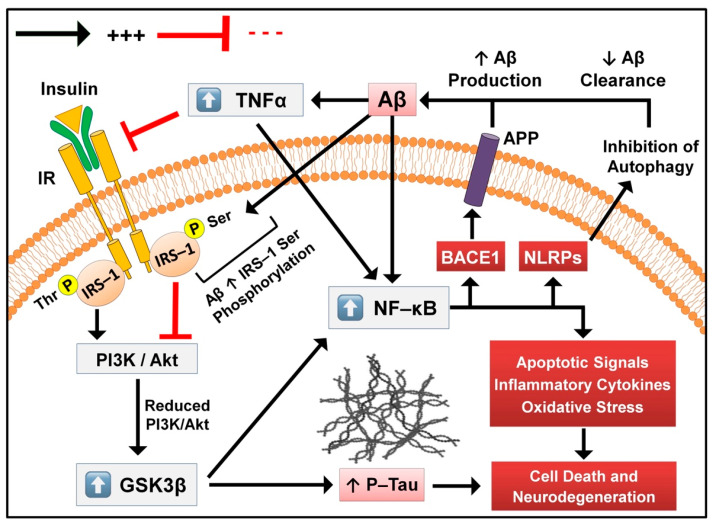
Schematic representation of some defective signaling pathways involved in the pathogenesis and progression of AD.

For instance, nuclear factor kappa b (NF-κB) activation was found to increase BACE1 expression and, in turn, Aβ production [25]. Moreover, triggers of canonical and/or alternative NF-κB signaling pathways, such as pro-inflammatory cytokines [26], pattern-recognition receptors (PRRs) [27], T- and B-lymphocytes [28] as well as tumor necrosis factor (TNF) receptor superfamily [29], were also found to correlate with the progression of AD; thus, highlighting the central regulatory role of NF-κB in relation to BACE1 expression. Interestingly, Aβ itself was also found to promote the activation of p65/p50 dimers of NF-κB and trigger the expression of pro-apoptotic genes [30]. Another example can be observed in insulin signaling, were Aβ impaired insulin-mediated responses via altering cellular levels and interactions of insulin receptors and low-density lipoprotein receptor-related protein-1 (LRP-1) in 3XTg-AD mice [31]. In addition, Aβ was observed to promote insulin resistance through promoting the serine phosphorylation of insulin receptor substrate-1 (IRS-1) [32], resulting in the reduction of phosphoinositide 3-kinase (PI3K) and protein kinase B (Akt) expression and enhanced glycogen synthase kinase-3 beta (GSK3β) activity [33]. Enhanced GSK3β activity was linked to the hyperphosphorylation of intracellular tau proteins leading to the development of neurofibrillary tangles [34]; accordingly, impaired insulin signaling was suggested to be a link between extra- and intracellular events in AD. GSK3β was also found to promote the activation of NF-κB and, in turn, sequential BACE1 expression and Aβ production [35]. Numerous other pathways have also been found to follow the same pattern, creating what appears to be a vicious cycle of endless events promoting the disease progression (Figure 2).

Such findings can illustrate the complex relationship between various signaling pathways and the progression of AD, which have led the scientific field to rethink whether Aβ is a major cause of AD or a consequence of multiple defective signaling pathways [36], especially that AD hallmarks appear at a late stage of the disease. All in all, this has promoted the emergence of numerous other hypotheses following the “Amyloid Cascade Hypothesis”, such the tau hypothesis [37], GSK3β hypothesis [38], oxidative stress hypothesis [39] amyloid-inflammatory hypothesis [40] and, ultimately, the multifactorial hypothesis of AD [41], which calls for taking into consideration the multifactorial nature of AD and the potential benefit of developing multi-target agents or combinational drug use. Needless to say, for years BACE1 seemed to be a tempting target for drug development and, indeed, numerous agents targeting BACE1 have emerged in experimental studies and others progressed into clinical trials. Findings regarding BACE1 inhibitors are discussed in the sections below.

## 3. BACE1 Inhibitors in Clinical Trials: What Happened?

Inspired by the “Amyloid Cascade Hypothesis” a number of pharmaceutical companies have dedicated time and efforts to develop pipelines and accelerate the introduction of small-molecule BACE1 inhibitors as potential disease modifying therapies for AD. Unfortunately, results of early clinical trials did not go in favor of BACE1 inhibitors, resulting in their discontinuation from production pipelines [4]. Below will include a discussion of the rise and fall of BACE1 inhibitors in clinical trials in order to dissect the factors that have led to the current status.

### 3.1. LY2811376

LY2811376 was the first small-molecule BACE1 inhibitor developed by Eli Lilly to enter clinical trials. In a translational phase I clinical trial (NCT00838084), completed in 2009, conducted to investigate the safety, pharmacodynamic and pharmacokinetic profiles of orally administered LY2811376, the drug demonstrated a significant decline of Aβ levels in the plasma and cerebrospinal fluid (CSF) in recruited subjects [42]. Unfortunately, further investigations were put on hold due to additional toxicological data reporting damage to eyes’ pigment epithelium in rats [43], and the drug was later discontinued by the company in favor of more potent and safer second generation BACE1 inhibitors. Despite the fact that LY2811376 was discontinued, it provided the first clinical evidence that BACE1 is a plausible target for AD. Currently, the drug use is restricted only to experimental studies as a selective BACE1 inhibitor [44,45].

### 3.2. LY2886721

LY2886721 is a second-generation BACE1 inhibitor developed by Eli Lilly, after its predecessor LY2811376, and the first BACE1 inhibitor to reach phase II clinical trials [43]. A total of six phase I clinical trials were conducted (NCT01534273, NCT01227252, NCT01133405, NCT01807026, NCT01775904 and NCT01367262), aiming to assess the safety, tolerability, pharmaco-kinetic and -dynamic profile as well as different formulations and dosing regimens of LY2886721 in healthy volunteers (only NCT01807026 included both healthy subjects and patients diagnosed with AD) [46]. Results of phase I trials have shown that LY2886721 is generally safe and well tolerated at different dosing regimens with dose-dependent central nervous system (CNS) disposition and the ability to target BACE1 and reduce multiple Aβ isoforms [47], and overall supported the introduction of LY2886721 into phase II. In 2012, a phase II trial (NCT01561430) was initiated to investigate the tolerability, efficacy, and pharmacodynamics of two different LY2886721 doses (15 mg and 35 mg) and included a total 128 AD patients with mild cognitive impairment or tested positive for Aβ deposition [48]. However, the trial was terminated as a number of participants exhibited abnormal biochemical liver test results that were independent (i.e., off-target) from BACE1 inhibition [49]. Therefore, Eli Lilly decided to halt further human studies with LY2886721, with a current discontinued status by the U.S. FDA for AD and patients with mild cognitive impairment. Later, May et al. (2015) elaborated more on the results of the phase II trial, where 4 out the 70 recruited subjects were reported to have abnormal liver enzyme elevations leading to the termination of the trial [50]. However, the relationship between LY2886721 and the abnormal liver enzyme elevations is still unclear, and the long-term clinical safety of BACE1 inhibitors should be further examined. Additionally, overcoming the relative non-selectivity of BACE1 vs. BACE2 inhibition is another factor that may promote the development of safer and more-effective drugs for AD [50]. Ultimately, safety concerns have doomed the use of LY2886721 in humans, yet it is still being utilized in experimental studies to provide clues for the potential effects of BACE1 inhibitors. For instance, in a recent study examining the effects of LY2886721 in PLB-4 mice, the drug has shown improved glucose homeostasis, hepatic gluconeogenesis, insulin sensitivity and beneficial effects on APP processing, supporting the potential utilization of BACE1 inhibitors for the treatment of type 2 diabetes mellitus-associated pathologies, especially in cases when diabetes is comorbid to AD [51].

### 3.3. RG7129 (RO5508887)

RG7129 is an orally administered BACE1 inhibitor manufactured by Roche [52]. In late 2011 and 2012, Roche launched three phase 1 clinical trials (NCT01461967, NCT01592331 and NCT01664143) to assess the safety, pharmacokinetics and pharmacodynamics of RG7129 in healthy participants [53]. However, in late 2013, Roche decided to discontinue the development of RG7129 with no official statement issued or results posted so far; however, liver toxicity was mentioned as a reason for the drug termination [54].

### 3.4. BI 1181181

BI 1181181 is a small-molecule BACE1 inhibitor that was initially discovered by Vitae Pharmaceuticals and clinically developed by Boehringer Ingelheim [55]. Preclinically, the drug showed impressive results in lowering Aβ levels in rats and guinea pigs [56,57]. In 2014, three phase I clinical trials were initiated to investigate the safety, tolerability and pharmacokinetic and pharmacodynamic properties of BI 1181181 in healthy participants [58]. Two of the three phase I trials (NCT02044406 & NCT02106247) were actually completed, and the results revealed that single doses of BI 1181181 were well tolerated with a substantial and sustained CSF Aβ reduction, and the pharmacokinetics were dose-proportional, not affected by food, and compatible with once-daily dosing [59,60]. The third phase I trial (NCT02254161) to primarily investigate the safety and tolerability of orally administered repeated rising doses of BI 1181181 (given once daily over 10 days) was terminated after reports of skin reactions among some of the participants [55]. In 2015, another phase I trial (NCT02345304), aiming to explore the effects of different doses of BI 1181181 on single-dose kinetics of midazolam, warfarin, omeprazole and digoxin, was withdrawn prior to recruitment. Eventually, the drug development was put to an end in favor a second-generation compound [55]. Currently, there is no mention of BI 1181181 in any further experimental or clinical studies.

### 3.5. JNJ-54861911 (Atabecestat)

JNJ-54861911 is a BACE1 inhibitor developed by Janssen. Over the past decade, the drug had a long history in clinical trials with promising findings and was able to progress and reach a phase II/III study [61]. Starting in 2013, Janssen initiated a series of phase I trials; 2013 (NCT01978548, NCT01827982 and NCT01887535), 2014 (NCT02152332, NCT02197884, NCT02211079, NCT02180269 and NCT02260700) and 2015 (NCT02611518, NCT02355561 and NCT02360657) [62]. Phase I trials were predominantly aimed at investigating the safety, tolerability, pharmacokinetics, pharmacodynamics, possible serious adverse events (i.e., effects on QT/QTc intervals), possible drug interactions (i.e., metformin and rosuvastatin) and food interaction (i.e., effect of a high-fat/high-caloric breakfast), of a single or multiple dosing regimens in healthy subjects [62]. Two of the aforementioned phase I trials (NCT01978548 and NCT02360657), aimed to investigate the safety, tolerability and pharmaco-kinetics, and -dynamics in patients with prodromal AD as well as CSF Aβ levels in asymptomatic subjects at risk for AD, respectively. Results of phase I clinical trials have shown that JNJ-54861911 was generally well tolerated and able to potently penetrate into the CNS and achieve high and stable reduction in Aβ levels. In addition, the trials reported, for the first time, a correlation between CSF BACE1 and its downstream marker Aβ_42_; however, the importance of routinely measuring BACE1 in daily clinical practice and AD clinical trials remains to be elucidated [63,64,65].

In parallel, Janssen started two phase II trials; 2014 (NCT02260674) and 2015 (NCT02406027), to assess the long-term safety and tolerability of different JNJ-54861911 doses in patients with early AD; in addition to, a 2015 phase II/III trial (NCT02569398) to evaluate the actual efficacy of JNJ-54861911 in slowing down the cognitive decline in Aβ-positive asymptomatic subjects with high AD risk [62]. Nevertheless, both of the 2015 studies were eventually terminated, and in 2018 Janssen announced the discontinuation of the drug due to reports of liver toxicity in test subjects [66]. The preliminary and full results of the trials were later published and revealed that JNJ-54861911 treatment did not show any benefit over placebo, caused an elevation of liver enzymes and showed a trend towards declines in cognition with evidence of reversibility after 6 months off treatment [67,68,69]. As a last attempt, a 2018 trial (NCT03587376) was conducted to explore T-cell mediated inflammatory immune response in subjects who were previously administered the drug, as a suggested pathway mediating JNJ-54861911 or its metabolites-induced liver toxicity; however, the trial was also terminated. A liver biopsy from one of the volunteers who experienced an elevation in liver enzymes showed signs of inflammation with an increase in T and B-cell infiltrates as well as hepatocyte death [70]. Later results revealed the detection of JNJ-54861911 metabolite-responsive T-cell clones in patients who experienced liver toxicity, which indicates the presence of an immune-based mechanism for the observed liver enzyme elevations [71]. In 2022, JNJ-54861911 holds a discontinued status for AD and without further use in clinical or experimental studies.

### 3.6. LY3314814 (AZD3293, Lanabecestat)

LY3314814 is a BACE1 and BACE2 inhibitor developed by a joint collaboration between AstraZeneca and Eli Lilly [72]. Preclinical data from various animal models showed promising outcomes and supported the progress of LY3314814 into clinical trials [73,74]. Between 2012 and 2017, numerous phase I trials were conducted to assess the safety, tolerability, drug interactions and pharmaco-kinetic and dynamic profiles of various LY3314814 doses and dosing regimens [75,76]. Results from phase I trials found that LY3314814 was generally safe and well-tolerated with a robust reduction in plasma and CSF Aβ levels among different drug formulations [77,78,79]. Directly from phase I to phase III, completely skipping phase II, a 2014 phase II/III trial (NCT02245737), referred to as “AMARANTH” trial, was conducted to investigate the safety and efficacy of LY3314814 for a period of 104 weeks in the treatment of early AD. In 2016, the “AMARANTH” trial was followed by another two phase III studies (NCT02972658 and NCT02783573), with a total of just over 4300 participants enrolled in the three trials. However, after an independent assessment conducted in 2018, all of the three phase III trials were terminated as the trials were not likely to meet the primary endpoints upon by completion and, in turn, trials were put to an end for futility [80,81,82]. The phase III trials results were later published and confirmed the findings of the independent assessment, as LY3314814 treatment did not slow down the cognitive/functional decline or alter the disease progression and was actually associated with cognitive worsening as well as brain volume reduction [83,84,85]. Currently, LY3314814 holds a discontinued status for AD.

### 3.7. MK-8931 (MK-8931-009, Verubecestat)

MK-8931 is a small-molecule BACE1 and BACE2 inhibitor developed by Merck [86]. Three phase I clinical trials (NCT01496170, NCT01537757 and NCT02910739) were conducted to assess the safety, tolerability and pharmaco-kinetics and dynamics of MK-8931. Results of phase I studies illustrated a potential for MK-8931 in the treatment of AD, as MK-8931 was found to be generally well tolerated and was able to reduce mean CSF concentrations of the Aβ proteins: Aβ_40_, Aβ_42_, and soluble β fragment of APP [87,88]. In 2012, Merck started the “EPOCH” trial (NCT01739348), initially as a phase II that was expanded to phase III, enrolling over 2000 participants to investigate the efficacy and safety of MK-8931 over an intended period of 78 weeks in mild to moderate AD [89]. Followed by another phase III study, the “APECS” trial (NCT01953601), enrolling 1454 subjects to examine the safety and efficacy of MK-8931 in prodromal AD [90]. However, both trials; “EPOCH” and “APECS” were terminated. The published results revealed no benefit of MK-8931 in mitigating cognitive/functional decline in patients with mild-to-moderate AD and it even associated with cognitive worsening, brain volume loss, and multiple treatment-related adverse events, including falls and injuries, suicidal ideation, weight loss, sleep disturbance, skin rash and hair color change; however, MK-8931 was not associated with adverse effects on retinal thickness and verbal fluency tasks showed some improvement [84,91,92,93,94,95]. In 2022, MK-8931 holds a discontinued status for AD and was unlisted from Merck’s pipeline.

### 3.8. E2609 (Elenbecestat)

E2609 is a small-molecule BACE inhibitor, with more selectivity (3.53-fold) towards BACE1 compared to BACE2, and clinically developed by both Biogen and Eisai Co. Ltd. [96]. Preclinical data showed that E2609 was able to significantly lower Aβ levels and was not associated with hypopigmentation as seen in other BACE inhibitors, such as MK-8931, which is attributed to its selectivity towards BACE1 than BACE2 [97]. Between 2011 and 2017, ten phase I clinical trials (NCT01975636, NCT02859207, NCT03055962, NCT01511783, NCT02207790, NCT01600859, NCT01716897, NCT01294540, NCT02222324 and NCT02055703) were conducted to assess the safety, tolerability, pharmaco-kinetics and -dynamics and drug- and food-interactions of different doses and regimens of E2609. Results of some of phase I trials were published, which highlighted that E2609 was generally well tolerated and did not display treatment-emergent adverse events or any clinically important effects on vital signs or electrocardiogram (ECG) and did not require restrictions or dose adjustments when co-administered with CYP3A inhibitors [98,99,100]. In December 2014, a phase II trial (NCT02322021) was launched to evaluate its safety and efficacy in participants with mild cognitive impairment with AD, prodromal AD or mild to moderate dementia due to AD [101]. Early results of the phase II trial revealed that E2609 was generally well tolerated, had no unexpected safety concerns emerged, was not discontinued by any subject due to liver toxicity and caused a statistically significant reduction in Aβ burden [102]. In parallel with the phase II trial, two multi-center phase III studies (NCT02956486 and NCT03036280), referred to as “MissionAD1” and “MissionAD2” respectively, were initiated in 2016 to evaluate the efficacy and safety of E2609 in patients with early AD [103,104]. However, in 2019 both phase III trials as well as the ongoing phase II trial were all terminated, because of an unfavorable risk-benefit ratio, no evidence of potential efficacy and worse adverse event profile of E2609 than placebo [101,103]. Some of the phase III results were later presented in the 2021 Alzheimer’s Association International Conference, highlighting that there was no evidence of treatment effectiveness in the early-terminated “MissionAD” program even in the very mild subjects [105]. In 2022, E2609 is discontinued for AD.

### 3.9. CNP-520 (Umibecestat)

CNP-520 is a BACE1 inhibitor, administered in capsule formulation, and jointly developed by Novartis and Amgen [106]. Early data showed that CNP520 was able to decrease brain and CSF Aβ levels and deposition in rats, dogs and APP-transgenic mice and demonstrated sufficient safety with no signs of depigmentation, retina, liver or cardiovascular toxicity [107]. In a multi-center phase II (NCT02576639) dose-ranging safety and tolerability study in 2015 in subjects above 60 years old, CNP520 was found to be generally safe and well tolerated and resulted in a robust dose-dependent CSF Aβ reduction [107]. Later on, two registered phase II/III trials (2015; NCT02565511 and 2017; NCT03131453), referred to as “Generation 1” and “Generation 2” respectively, were terminated in 2019 after early reports of safety issues with CNP-520; including, decline in cognition, brain atrophy and weight loss [108,109]. Nonetheless, these adverse events were found to be reversible with the follow-up monitoring after the discontinuation of the drug treatment [110]. Currently, CNP-520 is discontinued for AD.

### 3.10. LY3202626

LY3202626 is another BACE1 inhibitor by Eli Lilly [111]. Results from preclinical studies demonstrated the ability of LY3202626 to produce a concentration-dependent reduction of Aβ expression in PDAPP mice primary neuronal cultures; in addition, it reduced hippocampal and cortical Aβ and sAPPβ levels in PDAPP mice and beagle dogs following oral treatment [112,113]. Between 2014 and 2017, three phase I trials (NCT02323334, NCT02555449 and NCT03023826) were conducted in healthy subjects [114]. The results of these phase I trials revealed that LY3202626 was generally well tolerated among all tested doses, reached maximum plasma concentration 3 h post admiration, was able to freely penetrate BBB, produced dose-dependent decline in both plasma and CSF Aβ_40_ and Aβ_42_ and its metabolism occurred primarily via O-demethylation and amide hydrolysis [115,116]. In June 2016, a phase II clinical trial (NCT02791191), referred to as the “NAVIGATE-AD” trial, was conducted to evaluate the safety and effects of LY3202626 on brain tau in patients with mild AD [117]. Unfortunately, the “NAVIGATE-AD” trial was terminated due to the low likelihood of identifying a statistically significant treatment effect. The results were later published showing that LY3202626, while generally well tolerated, had no significant effect when compared to placebo [118]. In the same year, LY3202626 was also dropped from another phase II trial (NCT03367403), referred to as the “TRAILBLAZER-ALZ”, conducted to evaluate both LY3202626 and Donanemab, a monoclonal antibody against Aβ, in early symptomatic AD patients [111,119]. In 2022, LY3202626 is discontinued for AD.

### 3.11. PF-06751979

PF-06751979 is a small-molecule BACE1 inhibitor, with high BACE1 selectivity compared to BACE2, that was developed by Pfizer [120,121]. Three phase I trials (2015; NCT02509117, 2016; NCT02793232 and 2017; NCT03126721) were conducted to evaluate the safety, tolerability and pharmaco-kinetics and dynamics of PF-06751979 [122]. The published results of phase I trials indicate that PF-06751979 was generally well tolerated at all tested doses and reported adverse events were mild to moderate. The pharmacokinetic parameters remained consistent across once daily dosing regimens with no notable food effects and pharmacodynamic analysis showed a concentration-dependent reduction in CSF and plasma Aβ (the greatest reductions were observed with 275 mg once-daily dosing) [122]. These results were in favor of further clinical development. Nevertheless, in January 2018, Pfizer announced that they were ending their development lines in neurology, including PF-06751979, and thus the drug did not enter further phase II or III trials [120]. As of 2022, the drug holds the discontinued status for AD.

### 3.12. CTS21166

CTS21166 is a BACE1 inhibitor developed by CoMentis in partnership with Astellas Pharma [123]. In APP-transgenic mice, CTS21166 was able to significantly lower Aβ level and deposition [123]. Only one registered phase I trial (NCT00621010) was found, conducted back in 2008 to evaluate the safety and tolerability of CTS21166 in healthy male volunteers [124]. However, neither the results of the trial nor records of further human studies were found.

### 3.13. HPP854

HPP854 is a BACE1 inhibitor developed by High Point Pharmaceuticals, LLC. [125]. No record of preclinical studies investigating HPP854 was found. Only one 2011 registered phase I trial (NCT01482013) was found; conducted to evaluate the safety, tolerability and pharmaco-kinetic and dynamic relationships as well as CSF and plasma concentrations [125]. Nonetheless, neither the results of the trial were posted/published nor were records of further human studies found.

## 4. Discussion and Future Perspectives

The past decade was full of hopes and dreams of finding disease-modifying therapies for AD, especially with a number of BACE1 inhibitors advancing in clinical trials; however, what happened can be described as a graveyard for BACE1 inhibitors. In addition, it appears that pharmaceutical companies have actually abandoned BACE1 inhibitors for good, as not a single BACE1 inhibitor is currently listed in any company’s pipeline for experimental or clinical development.

The overwhelming failure of BACE1 inhibitors and its sister group, γ-secretase inhibitors, [126] has further supported the notion of abandoning, as described, the outdated “Amyloid Cascade Hypothesis” as a whole, since targeting Aβ production did not initially seem to be a feasible option for AD when it was put to the test in human studies [127]. Nevertheless, the hypothesis itself was not completely abandoned and various other Aβ-related drugs have progressed into clinical trials. For instance, Aβ anti-aggregates, Aβ transport enhancers, Aβ vaccines and passive immunization therapies against Aβ, were all candidates for AD and progressed into clinical studies [4]. Thereafter, and on its 30th anniversary, the “Amyloid Cascade Hypothesis” with its massive impact has celebrated its first success with the FDA approval of Aducanumab (sold under the brand name Aduhelm^®^) [128]. Aducanumab is an anti-Aβ monoclonal antibody, which was approved for the use in AD patients with mild cognitive impairment or mild dementia stage of the disease [129]. Aducanumab, a human immunoglobulin gamma 1 (IgG1) monoclonal antibody, exerts its mechanism of action via crossing the BBB and selectively binding aggregated soluble oligomers and insoluble fibril conformations of Aβ plaques in the brain [129]. However, much like BACE1 inhibitors, anti-Aβ antibodies had a long history of failures in clinical trials prior to the success of Aducanumab. For example, AAB-001 (Bapineuzumab), AAB-003 (PF-05236812), GSK933776 and LY2062430 (Solanezumab) all have failed due to lack of efficacy [130]. On the other hand, Aducanumab succeeded and other anti-Aβ antibodies are still in clinical phases holding hopes for more AD therapies.

Projecting the success of the anti-Aβ antibody Aducanumab to the case of BACE1 inhibitors may intrigue the revitalization of a newer generation BACE1 inhibitors that are efficacious and able to tackle safety issues of the older generation. For instance, one of the issues (i.e., hypopigmentation) was actually solved through selectively inhibiting BACE1 without interaction with BACE2. Until then, extensive research should be done to determine the underlying mechanisms linking BACE1 inhibition to the previously reported adverse events, and whether solely inhibiting BACE1 would be sufficient enough to produce sustained efficacy.

While this review has focused mainly on one research arm of AD therapies, it is worth to note and appreciate the ongoing extensive experimental and clinical research efforts aiming for the development of novel AD therapies, including medicinal plants and natural products [131], tau-targeted therapies [132], neurotransmitter modulators [133], neurotrophic factors [134], insulin sensitizers [135], dietary supplementation [136], procedural interventions [137] and numerous other therapy approaches.

## Data Availability

Not applicable.

## References

[1] Scheltens P., De Strooper B., Kivipelto M., Holstege H., Chételat G., Teunissen C.E., Cummings J., van der Flier W.M. (2021). Alzheimer’s disease. Lancet.

[2] World Alzheimer Report 2022. Life after Diagnosis: Navigating Treatment, Care and Support. https://www.alzint.org/resource/world-alzheimer-report-2022/.

[3] Hippius H., Neundörfer G. (2003). The discovery of Alzheimer’s disease. Dialogues Clin. Neurosci..

[4] Bazzari F.H., Abdallah D.M., El-Abhar H.S. (2019). Pharmacological interventions to attenuate Alzheimer’s disease progression: The story so far. Curr. Alzheimer Res..

[5] Ju Y., Tam K.Y. (2022). Pathological mechanisms and therapeutic strategies for Alzheimer’s disease. Neural Regen. Res..

[6] Wong K.H., Riaz M.K., Xie Y., Zhang X., Liu Q., Chen H., Bian Z., Chen X., Lu A., Yang Z. (2019). Review of current strategies for delivering Alzheimer’s disease drugs across the blood-brain barrier. Int. J. Mol. Sci..

[7] Armstrong R.A. (2019). Risk factors for Alzheimer’s disease. Folia Neuropathol..

[8] Chai B., Gao F., Wu R., Dong T., Gu C., Lin Q., Zhang Y. (2019). Vitamin D deficiency as a risk factor for dementia and Alzheimer’s disease: An updated meta-analysis. BMC Neurol..

[9] Dolatshahi M., Salehipour A., Saghazadeh A., Sanjeari Moghaddam H., Aghamollaii V., Fotouhi A., Tafakhori A. (2022). Thyroid hormone levels in Alzheimer disease: A systematic review and meta-analysis. Endocrine.

[10] Braak H., de Vos R.A., Jansen E.N., Bratzke H., Braak E. (1998). Neuropathological hallmarks of Alzheimer’s and Parkinson’s diseases. Prog. Brain Res..

[11] Hardy J.A., Higgins G.A. (1992). Alzheimer’s disease: The amyloid cascade hypothesis. Science.

[12] Yan R., Vassar R. (2014). Targeting the β secretase BACE1 for Alzheimer’s disease therapy. Lancet Neurol..

[13] Müller U.C., Deller T., Korte M. (2017). Not just amyloid: Physiological functions of the amyloid precursor protein family. Nat. Rev. Neurosci..

[14] Kojro E., Fahrenholz F. (2005). The non-amyloidogenic pathway: Structure and function of α-secretases. Subcell Biochem..

[15] Sun X., Chen W.D., Wang Y.D. (2015). β-Amyloid: The key peptide in the pathogenesis of Alzheimer’s disease. Front. Pharmacol..

[16] Sathya M., Premkumar P., Karthick C., Moorthi P., Jayachandran K.S., Anusuyadevi M. (2012). BACE1 in Alzheimer’s disease. Clin. Chim. Acta.

[17] Sun X., Tong Y., Qing H., Chen C.H., Song W. (2006). Increased BACE1 maturation contributes to the pathogenesis of Alzheimer’s disease in Down syndrome. FASEB J..

[18] Chávez-Gutiérrez L., Szaruga M. (2020). Mechanisms of neurodegeneration—Insights from familial Alzheimer’s disease. Semin. Cell Dev. Biol..

[19] Rauf A., Badoni H., Abu-Izneid T., Olatunde A., Rahman M.M., Painuli S., Semwal P., Wilairatana P., Mubarak M.S. (2022). Neuroinflammatory markers: Key indicators in the pathology of neurodegenerative diseases. Molecules.

[20] Bazzari F.H., Abdallah D.M., El-Abhar H.S. (2019). Chenodeoxycholic acid ameliorates AlCl3-induced Alzheimer’s disease neurotoxicity and cognitive deterioration via enhanced insulin signaling in rats. Molecules.

[21] Teixeira J.P., de Castro A.A., Soares F.V., da Cunha E.F., Ramalho T.C. (2019). Future therapeutic perspectives into the Alzheimer’s disease targeting the oxidative stress hypothesis. Molecules.

[22] Ma C., Hong F., Yang S. (2022). Amyloidosis in Alzheimer’s disease: Pathogeny, etiology, and related therapeutic directions. Molecules.

[23] Schultz S.A., Gordon B.A., Mishra S., Su Y., Perrin R.J., Cairns N.J., Morris J.C., Ances B.M., Benzinger T.L. (2018). Widespread distribution of tauopathy in preclinical Alzheimer’s disease. Neurobiol. Aging.

[24] Bazzari A.H., Parri H.R. (2019). Neuromodulators and long-term synaptic plasticity in learning and memory: A steered-glutamatergic perspective. Brain Sci..

[25] Qiao A., Li J., Hu Y., Wang J., Zhao Z. (2021). Reduction BACE1 expression via suppressing NF-κB mediated signaling by Tamibarotene in a mouse model of Alzheimer’s disease. IBRO Neurosci. Rep..

[26] Taipa R., das Neves S.P., Sousa A.L., Fernandes J., Pinto C., Correia A.P., Santos E., Pinto P.S., Carneiro P., Costa P. (2019). Proinflammatory and anti-inflammatory cytokines in the CSF of patients with Alzheimer’s disease and their correlation with cognitive decline. Neurobiol. Aging.

[27] Keogh C.E., Rude K.M., Gareau M.G. (2021). Role of pattern recognition receptors and the microbiota in neurological disorders. J. Physiol..

[28] Cao W., Zheng H. (2018). Peripheral immune system in aging and Alzheimer’s disease. Mol. Neurodegener..

[29] Ortí-Casañ N., Wu Y., Naudé P.J., De Deyn P.P., Zuhorn I.S., Eisel U.L. (2019). Targeting TNFR2 as a novel therapeutic strategy for Alzheimer’s disease. Front. Neurosci..

[30] Sun E., Motolani A., Campos L., Lu T. (2022). The pivotal role of NF-KB in the pathogenesis and therapeutics of Alzheimer’s disease. Int. J. Mol. Sci..

[31] Gali C.C., Fanaee-Danesh E., Zandl-Lang M., Albrecher N.M., Tam-Amersdorfer C., Stracke A., Sachdev V., Reichmann F., Sun Y., Avdili A. (2019). Amyloid-beta impairs insulin signaling by accelerating autophagy-lysosomal degradation of LRP-1 and IR-β in blood-brain barrier endothelial cells in vitro and in 3XTg-AD mice. Mol. Cell. Neurosci..

[32] Rahman S.O., Panda B.P., Parvez S., Kaundal M., Hussain S., Akhtar M., Najmi A.K. (2019). Neuroprotective role of astaxanthin in hippocampal insulin resistance induced by Aβ peptides in animal model of Alzheimer’s disease. Biomed. Pharmacother..

[33] Zheng M., Wang P. (2021). Role of insulin receptor substance-1 modulating PI3K/Akt insulin signaling pathway in Alzheimer’s disease. 3 Biotech.

[34] Toral-Rios D., Pichardo-Rojas P.S., Alonso-Vanegas M., Campos-Peña V. (2020). GSK3β and tau protein in Alzheimer’s Disease and epilepsy. Front. Cell. Neurosci..

[35] Chauhan N., Paliwal S., Jain S., Verma K., Paliwal S., Sharma S. (2022). GSK-3β and its Inhibitors in Alzheimer’s Disease: A Recent Update. Mini Rev. Med. Chem..

[36] Wang S., Mims P.N., Roman R.J., Fan F. (2016). Is beta-amyloid accumulation a cause or consequence of Alzheimer’s disease?. J. Alzheimer’s Park. Dement..

[37] Maccioni R.B., Farías G., Morales I., Navarrete L. (2010). The revitalized tau hypothesis on Alzheimer’s disease. Arch. Med. Res..

[38] Hooper C., Killick R., Lovestone S. (2008). The GSK3 hypothesis of Alzheimer’s disease. J. Neurochem..

[39] Padurariu M., Ciobica A., Lefter R., Lacramioara Serban I., Stefanescu C., Chirita R. (2013). The oxidative stress hypothesis in Alzheimer’s disease. Psychiatr. Danub..

[40] Bivona G., Iemmolo M., Piccoli T., Agnello L., Lo Sasso B., Ciaccio M., Ghersi G. (2022). High Cerebrospinal Fluid CX3CL1 Levels in Alzheimer’s Disease Patients but Not in Non-Alzheimer’s Disease Dementia. J. Clin. Med..

[41] Gong C.X., Liu F., Iqbal K. (2018). Multifactorial hypothesis and multi-targets for Alzheimer’s disease. J. Alzheimer’s Dis..

[42] Martenyi F., Lowe S., Dean R.A., Monk S.A., Gonzales C.R., Friedrich S., May P.C., Audia J.E., Citron M., LaBell E.S. (2010). P4-088: Central and Peripheral Pharmacokinetic and Pharmacodynamic Effects of the β-site APP Cleavage Enzyme (BACE1) Inhibitor LY2811376 In Humans. Alzheimer’s Dement..

[43] LY2886721. https://www.alzforum.org/therapeutics/ly2886721.

[44] Reising N.C., Day T.A., Hole J.T., Tingley III F.D., Gonzalez-DeWhitt P.A., Mergott D.J., McKinzie D.L., Demattos R.B., Hayashi M.L., Riddell D.R. (2018). P1-114: Measurement of endogenous mouse tau in cerebrospinal fluid from aged Pdapp mice following treatment with Ab-lowering compounds. Alzheimer’s Dement..

[45] Sun Q., Liu F., Zhao J., Wang P., Sun X. (2022). Cleavage of Kv2. 1 by BACE1 decreases potassium current and reduces neuronal apoptosis. Neurochem. Int..

[46] ClinicalTrials.gov. https://clinicaltrials.gov/ct2/results?cond=LY2886721&term=&cntry=&state=&city=&dist=.

[47] Martenyi F., Dean R.A., Lowe S., Nakano M., Monk S., Willis B.A., Gonzales C., Mergott D., Leslie D., May P. (2012). BACE inhibitor LY2886721 safety and central and peripheral PK and PD in healthy subjects (HSs). Alzheimer’s Dement..

[48] Study of LY2886721 in Mild Cognitive Impairment Due to Alzheimer’s Disease or Mild Alzheimer’s Disease. https://clinicaltrials.gov/ct2/show/NCT01561430?cond=LY2886721&draw=1&rank=6.

[49] Dong Y., Li X., Cheng J., Hou L. (2019). Drug development for Alzheimer’s disease: Microglia induced neuroinflammation as a target?. Int. J. Mol. Sci..

[50] May P.C., Willis B.A., Lowe S.L., Dean R.A., Monk S.A., Cocke P.J., Audia J.E., Boggs L.N., Borders A.R., Brier R.A. (2015). The potent BACE1 inhibitor LY2886721 elicits robust central Aβ pharmacodynamic responses in mice, dogs, and humans. J. Neurosci..

[51] Dekeryte R., Franklin Z., Hull C., Croce L., Kamli-Salino S., Helk O., Hoffmann P.A., Yang Z., Riedel G., Delibegovic M. (2021). The BACE1 inhibitor LY2886721 improves diabetic phenotypes of BACE1 knock-in mice. Biochim. Biophys. Acta Mol. Basis Dis..

[52] RG7129. https://www.alzforum.org/therapeutics/rg7129.

[53] ClinicalTrials.gov. https://clinicaltrials.gov/ct2/results?cond=RO5508887&term=&cntry=&state=&city=&dist=.

[54] Kumar D., Ganeshpurkar A., Kumar D., Modi G., Gupta S.K., Singh S.K. (2018). Secretase inhibitors for the treatment of Alzheimer’s disease: Long road ahead. Eur. J. Med. Chem..

[55] BI 1181181. https://www.alzforum.org/therapeutics/bi-1181181.

[56] Hobson S., Lenter M.C., Sauer A., Fuchs K., Lala D.S., Dillard L.W., Dorner-Ciossek C. (2015). P4-168: Effects of the bace1 inhibitor bi 1181181 and the anti-abeta antibody m266 on abeta in rat brain homogenate and CSF. Alzheimer’s Dement..

[57] Dorner-Ciossek C., Hobson S., Fuchs K., Sauer A., Bauer M., Morales-Ramos A., Venkatraman S., Dillard L.W., Kruk B., Howard L. (2015). P1-314: Pharmacological characterization of the new bace1 inhibitor bi 1181181. Alzheimer’s Dement..

[58] CliniclTrials.gov. https://clinicaltrials.gov/ct2/results?cond=BI+1181181&term=&cntry=&state=&city=&dist=.

[59] Borta A., Nicolas L., Kleiner O., Kammerer K.P., Schaible J., Pietzko K., Podhorna J., Dorner-Ciossek C., Scholpp J. (2015). P3-282: Single oral doses of the novel bace inhibitor bi 1181181 significantly reduce concentrations of cerebrospinal fluid amyloid-beta peptides in healthy subjects. Alzheimer’s Dement..

[60] Nicolas L., Kammerer K.P., Schaible J., Link J., Kleiner O., Borta A., Podhorna J., Scholpp J. (2015). P3-283: Pharmacokinetics, pharmacodynamics, and safety of the novel bace inhibitor bi1181181 after oral administration of single ascending doses in healthy subjects. Alzheimer’s Dement..

[61] Atabecestat. https://www.alzforum.org/therapeutics/atabecestat.

[62] ClinicalTrials.gov. https://clinicaltrials.gov/ct2/results?cond=JNJ-54861911&term=&cntry=&state=&city=&dist=.

[63] Timmers M., Van Broeck B., Ramael S., Slemmon J., De Waepenaert K., Russu A., Bogert J., Stieltjes H., Shaw L.M., Engelborghs S. (2016). Profiling the dynamics of CSF and plasma Aβ reduction after treatment with JNJ-54861911, a potent oral BACE inhibitor. Alzheimer’s Dement..

[64] Timmers M., Barão S., Van Broeck B., Tesseur I., Slemmon J., De Waepenaert K., Bogert J., Shaw L.M., Engelborghs S., Moechars D. (2017). BACE1 dynamics upon inhibition with a BACE inhibitor and correlation to downstream Alzheimer’s disease markers in elderly healthy participants. J. Alzheimer’s Dis..

[65] Timmers M., Streffer J.R., Russu A., Tominaga Y., Shimizu H., Shiraishi A., Tatikola K., Smekens P., Börjesson-Hanson A., Andreasen N. (2018). Pharmacodynamics of atabecestat (JNJ-54861911), an oral BACE1 inhibitor in patients with early Alzheimer’s disease: Randomized, double-blind, placebo-controlled study. Alzheimer’s Res. Ther..

[66] Liver Tox Ends Janssen BACE Program. https://www.alzforum.org/news/research-news/liver-tox-ends-janssen-bace-program.

[67] Henley D., Raghavan N., Sperling R., Aisen P., Raman R., Romano G. (2019). Preliminary Results of a Trial of Atabecestat in Preclinical Alzheimer’s Disease. N. Engl. J. Med..

[68] Novak G., Streffer J.R., Timmers M., Henley D., Brashear H.R., Bogert J., Russu A., Janssens L., Tesseur I., Tritsmans L. (2020). Long-term safety and tolerability of atabecestat (JNJ-54861911), an oral BACE1 inhibitor, in early Alzheimer’s disease spectrum patients: A randomized, double-blind, placebo-controlled study and a two-period extension study. Alzheimer’s Res. Ther..

[69] Sperling R., Henley D., Aisen P.S., Raman R., Donohue M.C., Ernstrom K., Rafii M.S., Streffer J., Shi Y., Karcher K. (2021). Findings of efficacy, safety, and biomarker outcomes of atabecestat in preclinical Alzheimer disease: A truncated randomized phase 2b/3 clinical trial. JAMA Neurol..

[70] De Jonghe S., Weinstock D., Aligo J., Washington K., Naisbitt D. (2021). Biopsy Pathology and Immunohistochemistry of a Case of Immune-Mediated Drug-Induced Liver Injury With Atabecestat. Hepatology.

[71] Thomson P.J., Kafu L., Meng X., Snoeys J., De Bondt A., De Maeyer D., Wils H., Leclercq L., Vinken P., Naisbitt D.J. (2021). Drug-specific T-cell responses in patients with liver injury following treatment with the BACE inhibitor atabecestat. Allergy.

[72] Lanabecestat. https://www.alzforum.org/therapeutics/azd3293.

[73] Eketjäll S., Janson J., Kaspersson K., Bogstedt A., Jeppsson F., Fälting J., Haeberlein S.B., Kugler A.R., Alexander R.C., Cebers G. (2016). AZD3293: A novel, orally active BACE1 inhibitor with high potency and permeability and markedly slow off-rate kinetics. J. Alzheimer’s Dis..

[74] Sims J.R., Selzler K.J., Downing A.M., Willis B.A., Aluise C.D., Zimmer J., Bragg S., Andersen S., Ayan-Oshodi M., Liffick E. (2017). Development review of the BACE1 inhibitor lanabecestat (AZD3293/LY3314814). J. Prev. Alzheimer’s Dis..

[75] ClinicalTrials.gov. https://clinicaltrials.gov/ct2/results?cond=AZD3293&term=&cntry=&state=&city=&dist=.

[76] ClinicalTrials.gov. https://clinicaltrials.gov/ct2/results?cond=Lanabecestat&term=&cntry=&state=&city=&dist=.

[77] Cebers G., Alexander R.C., Haeberlein S.B., Han D., Goldwater R., Ereshefsky L., Olsson T., Ye N., Rosen L., Russell M. (2017). AZD3293: Pharmacokinetic and pharmacodynamic effects in healthy subjects and patients with Alzheimer’s disease. J. Alzheimer’s Dis..

[78] Sakamoto K., Matsuki S., Matsuguma K., Yoshihara T., Uchida N., Azuma F., Russell M., Hughes G., Haeberlein S.B., Alexander R.C. (2017). BACE1 inhibitor lanabecestat (AZD3293) in a phase 1 study of healthy Japanese subjects: Pharmacokinetics and effects on plasma and cerebrospinal fluid Aβ peptides. J. Clin. Pharmacol..

[79] Ye N., Monk S.A., Daga P., Bender D.M., Rosen L.B., Mullen J., Minkwitz M.C., Kugler A.R. (2018). Clinical bioavailability of the novel BACE1 inhibitor lanabecestat (AZD3293): Assessment of tablet formulations versus an oral solution and the impact of gastric pH on pharmacokinetics. Clin. Pharmacol. Drug Dev..

[80] An Efficacy and Safety Study of Lanabecestat (LY3314814) in Early Alzheimer’s Disease (AMARANTH). https://clinicaltrials.gov/ct2/show/NCT02245737?cond=Lanabecestat&phase=12&draw=2&rank=1.

[81] A Study of Lanabecestat (LY3314814) in Early Alzheimer’s Disease Dementia. https://clinicaltrials.gov/ct2/show/NCT02972658?cond=Lanabecestat&phase=12&draw=2&rank=2.

[82] A Study of Lanabecestat (LY3314814) in Participants With Mild Alzheimer’s Disease Dementia (DAYBREAK-ALZ). https://clinicaltrials.gov/ct2/show/NCT02783573?cond=Lanabecestat&phase=12&draw=2&rank=3.

[83] Wessels A.M., Tariot P.N., Zimmer J.A., Selzler K.J., Bragg S.M., Andersen S.W., Landry J., Krull J.H., Downing A.M., Willis B.A. (2020). Efficacy and safety of lanabecestat for treatment of early and mild Alzheimer disease: The AMARANTH and DAYBREAK-ALZ randomized clinical trials. JAMA Neurol..

[84] Wessels A.M., Lines C., Stern R.A., Kost J., Voss T., Mozley L.H., Furtek C., Mukai Y., Aisen P.S., Cummings J.L. (2020). Cognitive outcomes in trials of two BACE inhibitors in Alzheimer’s disease. Alzheimer’s Dement..

[85] Zimmer J.A., Shcherbinin S., Devous Sr M.D., Bragg S.M., Selzler K.J., Wessels A.M., Shering C., Mullen J., Landry J., Andersen S.W. (2021). Lanabecestat: Neuroimaging results in early symptomatic Alzheimer’s disease. Alzheimer’s Dement..

[86] Verubecestat. https://www.alzforum.org/therapeutics/verubecestat.

[87] Chris Min K., Dockendorf M.F., Palcza J., Tseng J., Ma L., Stone J.A., Kleijn H.J., Hodsman P., Masuo K., Tanen M. (2019). Pharmacokinetics and Pharmacodynamics of the BACE 1 Inhibitor Verubecestat (MK-8931) in Healthy Japanese Adults: A Randomized, Placebo-Controlled Study. Clin. Pharmacol. Ther..

[88] Forman M., Palcza J., Tseng J., Stone J.A., Walker B., Swearingen D., Troyer M.D., Dockendorf M.F. (2019). Safety, Tolerability, and Pharmacokinetics of the β-Site Amyloid Precursor Protein-Cleaving Enzyme 1 Inhibitor Verubecestat (MK-8931) in Healthy Elderly Male and Female Subjects. Clin. Transl. Sci..

[89] An Efficacy and Safety Trial of Verubecestat (MK-8931) in Mild to Moderate Alzheimer’s Disease (P07738) (EPOCH). https://clinicaltrials.gov/ct2/show/NCT01739348?cond=MK-8931&draw=2&rank=4.

[90] Efficacy and Safety Trial of Verubecestat (MK-8931) in Participants with Prodromal Alzheimer’s Disease (MK-8931-019) (APECS). https://clinicaltrials.gov/ct2/show/NCT01953601?cond=MK-8931&draw=2&rank=3.

[91] Egan M.F., Kost J., Tariot P.N., Aisen P.S., Cummings J.L., Vellas B., Sur C., Mukai Y., Voss T., Furtek C. (2018). Randomized trial of verubecestat for mild-to-moderate Alzheimer’s disease. N. Engl. J. Med..

[92] Egan M.F., Mukai Y., Voss T., Kost J., Stone J., Furtek C., Mahoney E., Cummings J.L., Tariot P.N., Aisen P.S. (2019). Further analyses of the safety of verubecestat in the phase 3 EPOCH trial of mild-to-moderate Alzheimer’s disease. Alzheimer’s Res. Ther..

[93] Egan M.F., Kost J., Voss T., Mukai Y., Aisen P.S., Cummings J.L., Tariot P.N., Vellas B., van Dyck C.H., Boada M. (2019). Randomized trial of verubecestat for prodromal Alzheimer’s disease. N. Engl. J. Med..

[94] Sur C., Kost J., Scott D., Adamczuk K., Fox N.C., Cummings J.L., Tariot P.N., Aisen P.S., Vellas B., Voss T. (2020). BACE inhibition causes rapid, regional, and non-progressive volume reduction in Alzheimer’s disease brain. Brain.

[95] Sergott R.C., Raji A., Kost J., Sur C., Jackson S., Locco A., Patel A., Furtek C., Mattson B., Egan M.F. (2021). Retinal Optical Coherence Tomography Metrics Are Unchanged in Verubecestat Alzheimer’s Disease Clinical Trial but Correlate with Baseline Regional Brain Atrophy. J. Alzheimer’s Dis..

[96] Elenbecestat. https://www.alzforum.org/therapeutics/elenbecestat.

[97] Moriyama T., Fukushima T., Kokate T., Albala B. (2017). [P3–037]: Preclinical studies with elenbecestat, a novel bace1 inhibitor, show no evidence of hypopigmentation. Alzheimer’s Dement..

[98] Albala B., Lai R.Y., Aluri J., Boyd P., Chang M.K., Dayal S., Ferry J., Rege B. (2017). [P2–003]: Elenbecestat pharmacokinetic drug-drug interactions indicated no dosage adjustments required for most concomitant treatments. Alzheimer’s Dement..

[99] Lai R.Y., Darpo B., Dayal S., Hall N., Chang M.K., Albala B., Ferry J., Rege B. (2017). [P1–043]: Elenbecestat, a novel oral bace inhibitor, has no clinically meaningful effect on qtc interval up to a supratherapeutic dose of 200 mg. Alzheimer’s Dement..

[100] Hayata N., Yasuda S., Kanekiyo M., Ito S., Yoshida M., Kawaguchi H., Lai R.Y., Kaplow J., Albala B., Luthman J. (2018). P1-040: Elenbecestat, a novel bace inhibitor, demonstrates similar pharmacokinetics and tolerability in japanese subjects with multiple dosings. Alzheimer’s Dement..

[101] Dose-Finding Study to Evaluate Safety, Tolerability, and Efficacy of E2609 in Participants with Mild Cognitive Impairment Due to Alzheimer’s Disease (Prodromal Alzheimer’s Disease) and Mild to Moderate Dementia Due to Alzheimer’s Disease. https://www.clinicaltrials.gov/ct2/show/NCT02322021?term=E2609&draw=3&rank=10.

[102] Lynch S.Y., Kaplow J., Zhao J., Dhadda S., Luthman J., Albala B. (2018). P4-389: Elenbecestat, E2609, a bace inhibitor: Results from a phase-2 study in subjects with mild cognitive impairment and mild-to-moderate dementia due to Alzheimer’s disease. Alzheimer’s Dement..

[103] A 24-Month Study to Evaluate the Efficacy and Safety of Elenbecestat (E2609) in Participants with Early Alzheimer’s Disease (MissionAD1). https://www.clinicaltrials.gov/ct2/show/NCT02956486?term=E2609&draw=3&rank=9.

[104] A Placebo-Controlled, Double-Blind, Parallel-Group, 24-Month Study with an Open-Label Extension Phase to Evaluate the Efficacy and Safety of Elenbecestat (E2609) in Subjects With Early Alzheimer’s Disease. https://fdaaa.trialstracker.net/trial/NCT03036280/.

[105] Irizarry M.C., Gee M., Roberts C., Giorgi L., Kanekiyo M., LeBlanc M., Putti K., Kaplow J., Dhadda S. (2021). Cognitive Outcomes in the Very Mild Subgroup in the Phase 3 Studies of Elenbecestat in Early Ad (Mission ad Program). In 2021 Alzheimer’s Association International Conference. https://alz.confex.com/alz/2021/meetingapp.cgi/Paper/57910.

[106] Umibecestat. https://www.alzforum.org/therapeutics/umibecestat.

[107] Neumann U., Ufer M., Jacobson L.H., Rouzade-Dominguez M.L., Huledal G., Kolly C., Lüönd R.M., Machauer R., Veenstra S.J., Hurth K. (2018). The BACE-1 inhibitor CNP 520 for prevention trials in Alzheimer’s disease. EMBO Mol. Med..

[108] A Study of CAD106 and CNP520 Versus Placebo in Participants at Risk for the Onset of Clinical Symptoms of Alzheimer’s Disease (GS1). https://www.clinicaltrials.gov/ct2/show/NCT02565511?term=CNP520&draw=2&rank=3.

[109] A Study of CNP520 Versus Placebo in Participants at Risk for the Onset of Clinical Symptoms of Alzheimer’s Disease (GS2). https://www.clinicaltrials.gov/ct2/show/NCT03131453?term=CNP520&draw=2&rank=1.

[110] Umibecestat-Driven Cognitive Decline Is Reversible. https://www.alzforum.org/news/conference-coverage/umibecestat-driven-cognitive-decline-reversible.

[111] LY3202626. https://www.alzforum.org/therapeutics/ly3202626.

[112] McKinzie D.L., May P.C., Boggs L.N., Yang Z., Brier R.A., Monk S.A., Willis B.A., Borders A.R., Winneroski L.L., Green S.J. (2016). P1-080: Nonclinical Pharmacological Characterization of the Bace1 Inhibitor LY3202626. Alzheimer’s Dement..

[113] Boggs L.N., May P.C., Yang Z., Brier R.A., Monk S.A., Borders A.R., Winneroski L.L., Green S.J., Mergott D.J., McKinzie D.L. (2016). P3-035: A Correlational Analysis of Exposure and Pharmacodynamic Effects of the Bace1 Inhibitor LY3202626 in PDAPP Mice Following Acute Oral Dosing. Alzheimer’s Dement..

[114] ClinicalTrials.gov. https://clinicaltrials.gov/ct2/results?cond=&term=LY3202626&cntry=&state=&city=&dist=.

[115] Willis B.A., Lowe S.L., Daugherty L.L., Dean R.A., English B., Ereshefsky L., Gevorkyan H., James D.E., Jhee S., Lin Q. (2016). P1-044: Pharmacokinetics, Pharmacodynamics, Safety, and Tolerability of LY3202626, a Novel Bace1 Inhibitor, in Healthy Subjects and Patients with Alzheimer’s Disease. Alzheimer’s Dement..

[116] Katyayan K., Yi P., Monk S., Cassidy K. (2020). Excretion, mass balance, and metabolism of [14C] LY3202626 in humans: An interplay of microbial reduction, reabsorption, and aldehyde oxidase oxidation that leads to an extended excretion profile. Drug Metab. Dispos..

[117] A Study of LY3202626 on Disease Progression in Participants With Mild Alzheimer’s Disease Dementia (NAVIGATE-AD). https://clinicaltrials.gov/ct2/show/NCT02791191?term=LY3202626&draw=2&rank=2.

[118] Lo A.C., Evans C.D., Mancini M., Wang H., Shcherbinin S., Lu M., Natanegara F., Willis B.A. (2021). Phase II (NAVIGATE-AD study) results of LY3202626 effects on patients with mild Alzheimer’s disease dementia. J. Alzheimer’s Dis. Rep..

[119] A Study of LY3002813 in Participants With Early Symptomatic Alzheimer’s Disease (TRAILBLAZER-ALZ). https://clinicaltrials.gov/ct2/show/NCT03367403?term=LY3202626&draw=2&rank=5.

[120] PF-06751979. https://www.alzforum.org/therapeutics/pf-06751979.

[121] O’Neill B.T., Beck E.M., Butler C.R., Nolan C.E., Gonzales C., Zhang L., Doran S.D., Lapham K., Buzon L.M., Dutra J.K. (2018). Design and synthesis of clinical candidate PF-06751979: A potent, brain penetrant, β-site amyloid precursor protein cleaving enzyme 1 (BACE1) inhibitor lacking hypopigmentation. J. Med. Chem..

[122] Qiu R., Ahn J.E., Alexander R., Brodney M.A., He P., Leurent C., Mancuso J., Margolin R.A., Tankisheva E., Chen D. (2019). Safety, tolerability, pharmacokinetics, and pharmacodynamic effects of PF-06751979, a potent and selective oral BACE1 inhibitor: Results from phase I studies in healthy adults and healthy older subjects. J. Alzheimer’s Dis..

[123] Mikulca J.A., Nguyen V., Gajdosik D.A., Teklu S.G., Giunta E.A., Lessa E.A., Tran C.H., Terak E.C., Raffa R.B. (2014). Potential novel targets for A lzheimer pharmacotherapy: II. Update on secretase inhibitors and related approaches. J. Clin. Pharm. Ther..

[124] Safety Study of CTS21166 to Treat Alzheimer Disease (CTS). https://clinicaltrials.gov/ct2/show/NCT00621010?cond=CTS-21166&draw=2&rank=1.

[125] Safety Study of HPP854 in Subjects with Mild Cognitive Impairment or a Diagnosis of Mild Alzheimer’s Disease. https://clinicaltrials.gov/ct2/show/NCT01482013?cond=HPP-854&draw=2&rank=1.

[126] De Strooper B., Chávez Gutiérrez L. (2015). Learning by failing: Ideas and concepts to tackle γ-secretases in Alzheimer’s disease and beyond. Annu. Rev. Pharmacol. Toxicol..

[127] Luczkowski M. (2016). “No screams and cries will convince us that white is white and black is black”, an ode to the defenders of amyloid cascade hypothesis of Alzheimer’s disease. Coord. Chem. Rev..

[128] Dunn B., Stein P., Cavazzoni P. (2021). Approval of aducanumab for Alzheimer disease—The FDA’s perspective. JAMA Intern. Med..

[129] Cummings J., Aisen P., Apostolova L.G., Atri A., Salloway S., Weiner M. (2021). Aducanumab: Appropriate use recommendations. J. Prev. Alzheimer’s Dis..

[130] Hung S.Y., Fu W.M. (2017). Drug candidates in clinical trials for Alzheimer’s disease. J. Biomed. Sci..

[131] Bazzari A.H., Bazzari F.H. (2018). Medicinal plants for Alzheimer’s disease: An updated review. J. Med. Plant. Stud..

[132] Zhu L., Xu L., Wu X., Deng F., Ma R., Liu Y., Huang F., Shi L. (2021). Tau-targeted multifunctional nanoinhibitor for Alzheimer’s disease. ACS Appl. Mater. Interfaces.

[133] Kandimalla R., Reddy P.H. (2017). Therapeutics of neurotransmitters in Alzheimer’s disease. J. Alzheimer’s Dis..

[134] Bazzari A.H., Bazzari F.H. (2022). BDNF Therapeutic Mechanisms in Neuropsychiatric Disorders. Int. J. Mol. Sci..

[135] Yang J.J. (2022). Brain insulin resistance and the therapeutic value of insulin and insulin-sensitizing drugs in Alzheimer’s disease neuropathology. Acta Neurol. Belg..

[136] Chauhan P.S., Yadav D., Arukha A.P. (2022). Dietary Nutrients and Prevention of Alzheimer’s disease. CNS Neurol. Disord. Drug Targets.

[137] Sagud M., Tudor L., Pivac N. (2021). Personalized treatment interventions: Nonpharmacological and natural treatment strategies in Alzheimer’s disease. Expert Rev. Neurother..

